# Secretory IgA dysfunction underlies poor prognosis in *Fusobacterium*-infected colorectal cancer

**DOI:** 10.1080/19490976.2025.2528428

**Published:** 2025-07-16

**Authors:** Ilseok Choi, Kyung-A Kim, Sang Cheol Kim, Donghwan Park, Ki Taek Nam, Jun Hyung Cha, Seungbyn Baek, Junha Cha, Hye-Yeong Jo, Minsun Jung, Melody Y. Zeng, Irina Matei, Susan Bullman, Joong Bae Ahn, Yoon Dae Han, Han Sang Kim, Insuk Lee

**Affiliations:** a Department of Biotechnology, College of Life Science and Biotechnology, Yonsei University, Seoul, Republic of Korea; b Yonsei Cancer Center, Division of Medical Oncology, Department of Internal Medicine, Yonsei University College of Medicine, Seoul, Republic of Korea; c Division of Healthcare and Artificial Intelligence, Department of Precision Medicine, National Institute of Health, Cheongju, Republic of Korea; d Department of Biomedical Sciences, Brain Korea 21 FOUR Project for Medical Science, Yonsei University College of Medicine, Seoul, Republic of Korea; e Department of Pathology, Yonsei University College of Medicine, Seoul, Republic of Korea; f Children’s Cancer and Blood Foundation Laboratories, Departments of Pediatrics, and Cell and Developmental Biology, Drukier Institute for Children’s Health, Meyer Cancer Center, Weill Cornell Medicine, New York, NY, USA; g Immunology, James P. Allison Institute, The University of Texas MD Anderson Cancer Center, Houston, TX, USA; h Division of Colorectal Surgery, Department of Surgery, Yonsei University College of Medicine, Seoul, Republic of Korea; i Graduate School of Medical Science, Brain Korea 21 FOUR Project, Yonsei University College of Medicine, Seoul, Republic of Korea; j DECODE BIOME Co., Ltd., Incheon, Republic of Korea

**Keywords:** Colorectal cancer, *Fusobacterium nucleatum*, secretory IgA, plasma cell development, tumor bacterial burden

## Abstract

*Fusobacterium nucleatum* (Fn) is commonly enriched in colorectal cancer (CRC) and associated with poor outcomes, though its mechanisms remain unclear. Our study investigated how Fn affects the tumor microenvironment through single-cell transcriptomic analyses of 42 CRC patient tissues, comparing Fn-positive and Fn-negative tumors. We discovered that Fn impairs IgA plasma cell development and secretory IgA (sIgA) production by disrupting communication with tumor-associated macrophages. Additional experiments in germ-free mice, together with our re-analysis of a publicly available single-cell RNA-seq data set from a CRC mouse model with an intact gut microbiome–both models having been orally gavaged with Fn–jointly validated the causal role of Fn in impairing sIgA induction. We identified a dysregulated IgA maturation (IGAM) module in Fn-positive patients, indicating compromised mucosal immunity and increased bacterial infiltration. This IGAM signature effectively stratified Fn-positive patients, suggesting potential for targeted therapeutic approaches. Our findings reveal that Fn disrupts sIgA production, increasing tumor microbial burden and worsening prognosis through chronic inflammation in Fn-positive CRC.

## Introduction

Colorectal cancer (CRC) is one of the most prevalent and deadly cancers worldwide.^1^ Recent studies have underscored the potential role of the gut microbiome in both the development and progression of CRC.^2–4^ Specifically, *Fusobacterium nucleatum* (Fn) sensu lato, an anaerobic bacterium typically found in the oral cavity, has been identified as a key contributor to the pathogenesis of CRC.^5–7^ Previous research has demonstrated that Fn is highly enriched in colorectal cancer tissue and is associated with poor prognosis.^8–10^ These studies also suggest that Fn may promote tumorigenesis through various mechanisms, including evasion of the immune system, induction of a pro-inflammatory microenvironment, and activation of oncogenic pathways.^3,11,12^

Despite these significant discoveries, how Fn interacts with the tumor microenvironment to influence CRC outcomes remains unclear. Unraveling this complexity requires an in-depth single-cell-level analysis of these interactions. Single-cell RNA sequencing (scRNA-seq) has emerged as a powerful tool for studying the heterogeneity of cell populations within tumors, providing high-resolution information on the gene expression profiles of individual cells.^13,14^ This method is particularly effective in identifying specific immune cell types associated with the presence of Fn in colorectal tumors, thereby illuminating the interactions between Fn and the systemic immune response.

In this study, we aimed to investigate the effects of Fn on the tumor microenvironment by analyzing scRNA-seq data from both Fn-positive and Fn-negative samples. Our single-cell transcriptome analysis, corroborated by spatial transcriptome analysis, revealed that the presence of Fn impedes the maturation of secretory IgA (sIgA) by disrupting the communication between IgA plasma cells and tumor-associated macrophages (TAMs) involved in IgA induction and dysregulating a co-regulatory gene module essential for sIgA maturation. The causal role of Fn in sIgA dysfunction was confirmed by disrupted IgA plasma cell and macrophage development in Fn-administered mice. Furthermore, we observed increased bacterial infiltration within tumors, suggesting that Fn infection compromises the protective function of sIgA, potentially impacting prognosis. Additionally, we found that the activity of co-regulatory genes for sIgA maturation can stratify Fn-positive CRC patients for survival. These findings may provide valuable clinical implications for the treatment of Fn-positive CRC patients.

## Materials and methods

### Sample preparation

Single-cell isolation was performed using a Human Tumor Dissociation Kit (Cat# 130–095–929, Miltenyi Biotec, USA) following the manufacturer’s instructions. Fresh surgical tissues were cut into small pieces ( < 1 mm^3^) and placed into a gentleMACS C Tube (Cat# 130–093–237, Miltenyi Biotec, USA) containing a mixture of Enzyme H, Enzyme R, and Enzyme A in RPMI 1640 medium. The tube was then processed on a gentleMACS Dissociator (Cat# 130–093–235, Miltenyi Biotec, USA) using the GentleMACS programs h_tumor_01 and h_tumor_02, each followed by a 30-minute incubation at 37°C with rotation.

The dissociated tissues were filtered through a 70 μm MACS SmartStrainer (Cat# 130–098–462, Miltenyi Biotec, USA) to achieve a single-cell suspension. The filtrate was centrifuged at 300 × *g* for 10 minutes at 4°C. The resultant cell pellet was resuspended in 4 mL of RPMI 1640 medium (Cat# 11875093, Thermo Fisher Scientific, USA), and dead cells were removed using Ficoll-Paque PLUS (Cat# 17–1440–03, GE Healthcare, USA). The cells were subsequently washed and resuspended in PBS supplemented with 0.1% bovine serum albumin (BSA, Cat# 15561020, Invitrogen, USA). Cell viability was assessed using the LIVE/DEAD™ Viability/Cytotoxicity Kit (Cat# L3224, Invitrogen, USA), and the single-cell suspension was further enriched for viability with a Dead Cell Removal Kit (Cat# 130–090–101, Miltenyi Biotec, USA).

### Single-cell RNA sequencing (scRNA-seq) data generation and analysis

We generated single-cell transcriptome profiles using the 10x Chromium Single Cell 5’ Gene Expression Dual Index library. Single-cell suspensions were processed on a Chromium system (10x Genomics, USA). For the discovery cohort consisting of 24 samples, we prepared 5’ scRNA-seq libraries using the Chromium Next GEM Single Cell 5′ Library and Gel Bead Kit v1.1 (PN-1000165). For the validation cohort of 18 samples, 3’ scRNA-seq libraries were generated using the Chromium Next GEM Single Cell 3ʹ v3.1 Library and Gel Bead Kit v3.1 (PN-1000121). The libraries were sequenced on an Illumina NovaSeq 6000 for the 5’ scRNA-seq and an Illumina HiSeq 2000 for the 3’ scRNA-seq. Sequencing reads were aligned to the human reference genome (GRCh38) using Cell Ranger software (v6.0.0).

For data processing, we utilized the Seurat R package (v4.0.0).^15^ Initially, cells expressing fewer than 200 features were removed. We then conducted cell quality control for each sample, evaluating mitochondrial read percentage, total read count, and feature count thresholds. Doublets, which represent two or more cells captured together in a single droplet, were identified and excluded using the DoubletFinder (v2.0.3) R package.^16^

The count matrix underwent log-normalization for read depth normalization via the *NormalizeData()* function with default parameters. We identified 3,000 variable features using the *FindVariableFeatures()* function with the selection method set to ‘vst’. T-cell receptor and immunoglobulin variable genes, known for patient-specific variances, were classified as “bad genes” and removed from the list of 3,000 variable features and all subsequent analysis stages.

The cell cycle score, which quantifies the extent to which a cell’s transcriptional program aligns with cells in the G1 or S/G2/M phase, was calculated using the *CellCycleScoring()* function. We then regressed this score against mitochondrial percentage while scaling the data with the *ScaleData()* function.

Principal Component Analysis (PCA) was performed using the *RunPCA()* function, inputting the previously determined variable features. We corrected for batch effects among samples by removing patient-specific signals with the *RunHarmony()* function from the Harmony (v1.0)^17^ R package, using the top 50 principal components (PCs) as input. A *knn* graph was constructed using the *FindNeighbors()* function with the corrected 50 PCs. Non-linear dimensional reduction was achieved through the *RunUMAP()* function using the same 50 coordinates.

Cell clustering was performed with the *FindClusters()* function at a resolution of 0.5. To identify different cell types, we annotated each cell cluster using marker genes. To ensure a clear immune cell profile, we removed clusters annotated as epithelial cells, endothelial cells, goblet cells, fibroblasts, and myofibroblasts, and those with high mitochondrial or cell-cycle scores or lacking marker gene expression. After filtering, we repeated the analysis with the remaining immune cells to refine the immune cell profile.

For detailed analysis at the cell subtype level, each cell type was processed individually. For B-cells and plasma cells, we employed 1,000 variable genes, ten PCs, and a resolution of 0.8, removing clusters lacking marker gene expression before repeating the processing steps. We identified a cluster of dendritic cells from macrophages and monocytes using ten PCs and a resolution of 0.8, annotating clusters expressing *CD1C* and *CLEC10A* as dendritic cells.

### Fusobacterium nucleatum *(Fn) tumor infection analysis via 16S rRNA sequencing*

Total genomic DNA from surgical tumor tissues was extracted using QIAamp® DNA Mini Kit (Qiagen, USA) following the manufacturer’s protocol. DNA concentration was measured with the Qubit dsDNA HS Assay Kit and Qubit Fluorometer (Invitrogen, USA). Library preparation for 16S rRNA gene sequencing followed the Illumina protocol. Amplicon PCR targeting the bacterial 16S rRNA V3-V4 region (primers Bakt_341F-805 R) was performed under the following conditions: 95°C for 3 min; 25 cycles of 95°C for 30 sec, 55°C for 30 sec, 72°C for 30 sec; final extension at 72°C for 5 min. PCR products were verified on an Agilent Bioanalyzer (DNA 1000 chip, USA) and quantified using a Qubit Fluorometer. Amplicons were purified using AMPure XP beads (Beckman Coulter, USA), followed by index PCR with Nextera® XT Index primers (Illumina, USA) under the same cycling conditions, except for 8 cycles. Indexed libraries were purified with AMPure XP beads, and library concentration was determined using the Quant-iT™ PicoGreen™ dsDNA Assay Kit (Invitrogen, USA). Sequencing was performed on the Illumina MiSeq platform (Macrogen, South Korea), generating 2 × 300 bp paired-end reads.

Raw paired-end reads were assembled using FLASH (v1.2.11)^18^ to merge overlapping sequences and improve quality. Assembled reads were length filtered with CD-HIT-OUT (v4.8.1),^19^ retaining sequences between 400–500 bp. Redundant sequences were clustered at 100% identity using CD-HIT-DUP, and chimeric sequences were removed. Secondary clusters were merged into primary clusters, and noise sequences were filtered out based on size thresholds. Non-chimeric representative reads were clustered into OTUs at 97% identity for species-level classification using a greedy algorithm. Taxonomic assignment of OTUs was performed with QIIME against the NCBI 16S rRNA database (version 20,211,127). Taxonomic abundance ratios were calculated, and samples were classified as Fn-positive if Fn exceeded 1% relative abundance.

### Cellular composition analysis using Pearson residual

To investigate proportional changes between single-cell groups (e.g., Fn-positive and Fn-negative samples) across cell types, we evaluated deviations in the observed cell count for each cell type from the expected cell count using *Pearson residual*:

$$\mathrm{Pearson}\;\mathrm{residual}\;(r_{\mathrm{ij}})=\frac{O_{\mathrm{ij}}-E_{\mathrm{ij}}}{\sqrt{E_{\mathrm{ij}}}},$$

where *i* and *j* represent indices for each group and cell type, respectively, and *O* and *E* represent the observed and expected cell counts, respectively. An expected cell count (*E*) for a group *i* of a cell type *j* was calculated by the following equation:

$$E_{\mathrm{ij}}=\frac{T_{i}}{T_{\mathrm{tot}}}\cdot T_{j},$$

where *T*_*tot*_, *T*_*i*_, *T*_*j*_ represent total cell count for the entire data set, total cell count for a group *i*, and total cell count for a subset *j*, respectively. The sign of the residual indicates the direction of the difference of the observed count from the expected count (i.e., positive for increase and negative for decrease compared to expected count). *Pearson residual* (*r*) follows an approximately normal distribution; thus, scores larger than 2.58 or smaller than −2.58 are significant by *p* < 0.01. For a highly conservative statistical test along with *Bonferroni* correction for multiple hypothesis test adjustment, we used a significance threshold of *p*-value = 0.00045 (*p*-value of 0.01 was divided by the number of subsets). Thus, we counted only *r* > 3.5 (increase) and *r* < −3.5 (decrease) for cell types with significant changes in their abundance between groups. The goodness of fit for all subsets was also evaluated by the chi-square statistic (*p*-value).

### Determination of Fn-infection status in TCGA samples

Unmapped reads from the TCGA-COAD RNA-seq bam files, which do not align to the human reference genome, are expected to potentially include tumor-infiltrating microbial reads. To minimize false positives, we used GATK PathSeq,^20^ an alignment-based algorithm, to filter out human reads by aligning them against the human reference genome. Subsequently, the remaining reads were aligned to the Human Reference Gut Microbiome (HRGM) database^21^ to obtain a microbial profile using the *PathSeqPipelineSpark* tool with default parameters (min-clipped-read-length = 31, min-score-identity = 0.9, identity-margin = 0.02). The TCGA samples exhibiting a normalized Fn score greater than 0.001—a threshold heuristically determined to maximize the effects of Fn on survival probability – were annotated as Fn-positive.

### Survival analysis for TCGA-COAD samples

The count matrix and metadata were collected from TCGA-COAD datasets using TCGAbiolinks R package (v2.25.2).^22^ We obtained clinical data from TCGA Pan-Cancer Clinical Data Resource (TCGA-CDR),^23^ and the samples without clinical data were filtered. To effectively elicit the mechanism of Fn affecting the tumor microenvironment, we only used TCGA data obtained from the right colon (ascending colon and cecum), where Fn infection affects survival probability most robustly. Samples with microsatellite instability-high (MSI-H) were excluded based on MSI status obtained from an external database,^24^ leaving 121 TCGA samples for analysis.

We used progress-free interval (PFI) information from TCGA-CDR as clinical data for survival analysis. Survival curves were estimated with the *survfit()* function in the survival (v3.2–7) R package using the Kaplan-Meier method.^25^ We used the *ggsurvplot()* function in the survminer (v0.4.9) R package to plot survival curves. The *p*-value was calculated through the log-rank test with default parameters of the *ggsurvplot()* function. In survival analysis with a combination of the Fn label with the group of each cell type, the pairwise *p*-value was calculated through the log-rank test with samples belonging to the groups.

### RNA velocity analysis and estimation of cell state transition probability

The spliced and unspliced counts in each cell from the scRNA-seq data were estimated using the *run10x()* function of the Velocyto package (v0.17.17).^26^ RNA velocity was analyzed separately for each cell type to compare differentiation patterns between the Fn groups, utilizing the Dynamo Python package (v1.0.0).^27^ This recently released tool addresses the fundamental limitations of traditional RNA velocity analysis by enhancing the measurement of RNA velocity and enabling the determination of transition probabilities between cell types. We adhered to the standard workflow outlined in the Dynamo user guide to estimate RNA velocity. The transition probability matrix was derived by annotating observations within the data object. To determine the transition probability to a specific cell type, we summed the transition probabilities from a given cell to all cells of that type, providing a clearer understanding of cellular dynamics across different conditions.

### Cell-cell interaction estimation

The CellChat (v1.6.1)^28^ R package was used to infer interactions between whole cell types. Subsequently, interactions between B-cells and myeloid cells were investigated. The default CellChat database was utilized, and only significant interactions in at least one Fn group were visualized; if the interactions were insignificant for a particular group, no dot was plotted on the dot plot. The biological roles of the ligand-receptor pairs involved in the IgA induction were annotated based on previous studies.^29,30^

### Spatial transcriptomic data analysis

We obtained publicly available 10x Visium data from CRC tumors located in the right colon.^31^ We aligned the raw fastq files to the same reference genome as the scRNA-seq data using 10x Genomics Space Ranger (v3.0.0). To label Fn infection status, we followed the same method described in the section of tumor microbiome profiling of TCGA samples, selecting samples with the highest detection of Fn reads as Fn-positive and those with none as Fn-negative for further analysis. We used Scanpy (v1.9.8)^32^ to perform the basic processing of the Visium data. The raw count matrix was normalized using *calculate_qc_metrics()* function followed by log1p transformation. To reduce noise in the Visium data, we excluded mitochondrial genes, immunoglobulin variant genes, and T cell receptor variant genes, consistent with the scRNA-seq data.

To infer cell type composition of each spot, we used Cell2location (v0.1.3)^33^ to build a reference model based on our scRNA-seq data. To accurately reflect the histological characteristics within the tumor, the reference model included both immune and stromal cells from the scRNA-seq data. The spatial mapping model training followed the tutorial with recommended parameters (N_cells_per_location = 5, detection_alpha = 20, max_epochs = 30000). We used q05_cell_abundance as a conservative estimate of cell abundance in each spot. For further analysis, we labeled each spot as containing a particular cell type if the Cell2location estimate for that cell type was higher than 0.1. To measure the distance between cell types, we collected the spots containing each cell type and calculated the distance between coordinates on a spot-by-spot basis. For measuring ligand-receptor interactions in the 10x Visium data, we used COMMOT (v0.0.3)^34^ with the optimal transport algorithm. For the ligand-receptor database, we utilized the CellChat DB.^28^ When scoring CCI between cell types, we only used the CCI scores assigned to spots where the Cell2location estimate was higher than 0.1 for each cell type.

### Trajectory-based gene clustering and differential expression analysis of B-cell

For trajectory-based analysis, B-cells and plasma cells were analyzed separately using the Slingshot R package (v2.2.0).^35^ This tool was utilized to construct a lineage trajectory from naïve B-cell to IgA-secreting plasma cell, reflecting the IgA maturation pathway. Following lineage construction, the tradeSeq R package (v1.8.0)^36^ was employed to identify differentially expressed genes between Fn groups associated with this IgA maturation lineage. We applied a negative binomial generalized additive model using the *fitGAM()* function from tradeSeq, incorporating the count matrix, pseudotime, and cell weight information (nknots = 6). The Fn labels of the cells were integrated into the model using the condition parameter of *fitGAM()* to fit a condition-specific smoother for each lineage. The *conditionTest()* function was then used to identify genes with distinct expression dynamics across Fn labels. Genes meeting the criteria of a p-value less than 0.05 and a waldStat value greater than 20 were selected, ensuring the exclusion of ribosomal and mitochondrial genes, often considered as ‘bad genes.’ For gene clustering, we used the *clusterExpressionPatterns()* function (nPoints = 20), and labeling was performed with the *primaryCluster()* function of the clusterExperiment R package (v2.14.0). A gene cluster containing marker genes of IgA-secreting plasma cells, including *PRDM1*—a known marker of plasma cell maturation – was selected.

We employed the singscore R package (v1.10.0)^37^ for robust rank-based expression scoring of the IGAM gene set in TCGA data. TCGA samples with gene set expression scores higher than their median were classified into IGAM-high activity groups. Samples with scores below the median were classified into IGAM-low activity groups for detailed analysis.

### Co-expression network analysis for prioritizing core genes of the IGAM module

Initially, we utilized the metacell^38^ R package to reduce the sparsity of scRNA-seq data and construct a robust co-expression network. We divided the scRNA-seq matrix of IgA plasma cells into two separate matrices for Fn-negative and Fn-positive cells to compare the co-expression networks between the two Fn groups. We refined the raw matrix by removing mitochondrial genes, ribosomal genes, and previously identified ‘bad genes’ such as *NEAT1*, *TMSB4X*, and *TMSB10*.^38^ Cells with fewer than 500 read counts were also excluded. Following a guided tutorial, we processed the data using recommended parameters to obtain the metacell expression matrix. We employed *mcell_gset_filter_varmean()* and *mcell_gset_filter_cov()* functions to select informative genes (T_vm = 0.08, T_tot = 100, T_top3 = 2), *mcell_add_cgraph_from_mat_bknn()* function to construct a balanced cell graph (K = 100), *mcell_coclust_from_graph_resamp()* (min_mc_size = 20, p_resamp = 0.75, n_resamp = 500) and *mcell_mc_from_coclust_balanced()* (K = 30, min_mc_size = 30, alpha = 2) functions to resample and generate the co-clustering graph, and *mcell_mc_split_filt()* (T_lfc = 3) function to remove outlier cells within the metacell. After obtaining the refined metacell expression matrix, we constructed a co-expression network by calculating the Pearson correlation coefficient (PCC) using the *cor()* function from the R stats package.

To prioritize genes in the IGAM module, we analyzed the PCC difference between Fn-negative and Fn-positive gene pairs. Gene pairs were sorted by PCC difference, and a two-sided Wilcoxon test was used to prioritize genes with significant differences. Genes with a p-value lower than 0.05 were selected as the core components of the IGAM module.

### Tumor microbiota analysis

To quantify the bacterial burden within tumor tissues, we analyzed read counts for tumor-infiltrating bacteria using GATK PathSeq. We obtained the number of unambiguous reads aligned to bacteria at the domain level. To adjust for sequencing depth bias, we normalized the bacterial read counts by dividing by the total number of reads in the original bam file, then multiplied by a scale factor of 1 million, and converted the results to a logarithmic scale.

For species-specific analyses, we employed a similar normalization method but increased the scale factor to 10 million to account for the typically smaller number of reads aligned to each species. This adjustment allows for a more precise comparison of tumor bacterial abundance between Fn groups.

To compare the IgA binding probabilities of tumor bacterial species between samples, we applied previously established binding probabilities to each species. We averaged probabilities obtained from multiple samples within each group. For consistent comparison, we adjusted the distribution of values by replacing infinite values with 20 and zero values with 0.0001, following the approach used in a previous study.

### Analysis of validation cohort tumor samples

For estimating cell-cell interactions, we employed CellPhoneDB (v2.0.0),^39^ and enhanced the analysis by integrating ligand-receptor information from SingleCellSignalR (v0.99.24)^40^ and CellTalkDB (v1.0)^41^ to expand our custom database.

RNA velocity measurements were conducted using Dynamo, following the procedures outlined previously. Additionally, we generated bulk RNA-seq data for the same samples. The Fn infection profile was determined from annotations based on 16S rRNA-seq data.

To align our RNA-seq data with the TCGA standards, the FASTQ files were processed using the STAR aligner, adhering to the RNA-seq alignment workflow provided by the NCI Genomic Data Commons (GDC).^42^ Tumor-infiltrating bacterial profiling was then carried out using PathSeq, following the same methods previously described. This also included a comparison of read abundance using the same normalization and analytical approaches.

### *Cultivation of* Fusobacterium nucleatum *(Fn)*

*Fusobacterium nucleatum* (ATCC 25586) was obtained from the American Type Culture Collection (ATCC, USA). Fn was cultured in Gifu Anaerobic Medium (GAM, Kisanbio, South Korea) broth under anaerobic conditions (20% CO_2_, 5% H_2_, 75% N_2_) at 37°C in a Coy chamber (Coy Laboratory Products, USA). For solid cultures, Fn was grown anaerobically on Brucella agar plates with 5% sheep blood at 37°C in the Coy chamber.

### Fn oral administration in germ-free (GF) mice

Six-week-old female GF C57BL/6 mice were used for all experiments. Ten mice were randomly divided into two groups (5 mice per group): a control group and an Fn group. All animal procedures were approved by the Institutional Animal Care and Use Committee (IACUC, #2022–0286) of Yonsei University College of Medicine and conducted in accordance with the Public Health Service Policy on Humane Care and Use of Laboratory Animals. Yonsei University is accredited by AAALAC International (#001071). GF mice were housed in individually ventilated cages (IVC) under controlled conditions (temperature: 22–23°C; light cycle: 12 h light/12 h dark). Sterile food and water were provided *ad libitum*.

Prior to oral gavage, GF mice were fasted for 2 hours. The Fn group received 1 × 10^9^ colony-forming units (CFU) of Fn in 200 µL of PBS, administered orally three times per week for 5 weeks. The control group received 200 µL of PBS alone on the same schedule. Mouse body weight was measured three times weekly until sacrifice. At the end of the experiment, the mice were sacrificed, and colon tissues were collected from both the control (*n* = 5) and Fn (*n* = 5) groups.

### Mouse colon and cecum tissue processing

Colon tissues, from proximal to distal sections, were cut open longitudinally and washed twice with ice-cold PBS. The tissues were then immersed in ice-cold RPMI-1640 medium for further processing and dissociation. The cecum was isolated from the gastrointestinal tract by horizontal dissection at the junctions between the cecum and the ileum, and between the cecum and the proximal colon. The cecum weight was measured for each group.

### Preparation of single-cell suspensions from mouse colon tissue for scRNA-seq library

Dissected colon tissues from PBS control (*n* = 5) and Fn-treated (*n* = 5) GF mice were pooled for each group. The tissues were cut into ~5 mm fragments and washed three times with ice-cold PBS. Pre-digestion was performed twice for 20 min at 37°C in Hanks’ Balanced Salt Solution (HBSS, Gibco, USA) without Ca^2 +^ and Mg^2 +^, supplemented with 5 mM EDTA, 5% fetal bovine serum (FBS), and 1 mM DTT, using a rotator mixer (Miltenyi Biotec, USA). The tissue fragments were then transferred to C-tubes (Cat# 130–096–334, Miltenyi Biotec, USA) containing HBSS with Ca^2 +^ and Mg^2 +^ (Gibco, USA) and an enzyme mix (enzymes D, R, and A) from the Mouse Lamina Propria Dissociation Kit (Cat# 130–097–410, Miltenyi Biotec, USA). Dissociation was performed at 37°C for 30 min using a gentleMACS Octo Dissociator with heaters (Cat# 130–134–029, Miltenyi Biotec, USA). Following incubation, ice-cold PB buffer (PBS with 0.5% bovine serum albumin) was added, and the samples were passed through a 100 μm cell strainer. The cell suspensions were centrifuged at 300 × *g* for 10 min at 4°C, and the pellet was resuspended in ice-cold PB buffer. The samples were filtered through a 70 μm cell strainer and rinsed once with PB buffer. Viable cells were purified using the Dead Cell Removal Kit (Cat# 130–090–101, Miltenyi Biotec, USA). Single-cell suspensions were prepared for scRNA-seq library construction using the 10X Chromium Next GEM Single Cell 5’ RNA Library v2 kits.

### Analysis of mouse-derived scRNA-seq data

The libraries were sequenced on an Illumina NovaSeq 6000, and sequencing reads were aligned to the mouse reference genome (GRCm39) using Cell Ranger (v9.0.0). The same processing steps–quality control, doublet removal, normalization, batch-effect correction, cell typing, and immune-cell filtering–were applied to both the germ-free mouse dataset and the publicly available CRC mouse dataset with a normal gut microbiome. B cells and IgA plasma cells were isolated and ordered along pseudotime using Monocle (v2.30.1),^43^ with DDRTree (v0.1.5) used for visualization. Spliced and unspliced read counts were generated with Velocyto, and RNA velocity was calculated with Dynamo.

In the colon tissue of the germ-free model, 2,593 B cells and IgA plasma cells were analyzed. Among these, 288 IgA plasma cells were assigned to state 6, while the remaining cells were divided evenly across five pseudotime states (approximately 461 cells per state) for RNA velocity analysis. In the tumor tissue of the CRC mouse model with a normal gut microbiome, 119 GC B cells, 1,158 B cells, and 603 IgA plasma cells were divided evenly into six pseudotime states (approximately 313 cells per state).

## Results

### Fn infection modifies the tumor immune landscape and is associated with reduced survival in CRC

**Figure 1.**
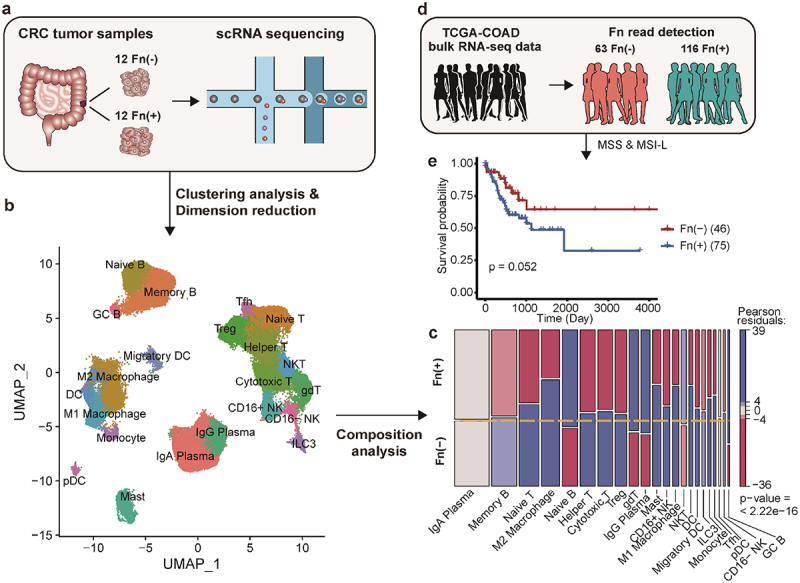
Single-cell RNA sequencing analysis shows Fn-associated changes in immune composition of CRC tumor.

We conducted single-cell transcriptomic analysis to thoroughly examine the cellular and molecular elements of the tumor microenvironment in CRC. We compared 12 Fn-positive and 12 Fn-negative patients, with Fn-infection status determined through 16S rRNA analysis. Our discovery cohort of 24 tumor samples was then subjected to scRNA-seq to assess cellular composition and gene expression profiles (Figure 1(a), Supplementary Table S1, S2a). Given the role of immune system in bacterial infections, we focused on changes in the immune microenvironment following Fn infection. After identifying 22 immune cell types from 109,204 cells (Figure 1(b), Supplementary Figure S1a, Supplementary Table S2b), we observed an unbiased distribution across patients, ensuring data integrity through proper pre-processing and batch corrections (Supplementary Figure S1b).

(a) Tumor tissues from 24 patients with CRC (12 Fn-positives and 12 Fn-negatives) were subjected to the single-cell RNA sequencing analysis. (b) UMAP visualization of immune cells from 24 patients after pre-processing and batch correction. A total of 22 immune cell types were obtained. (c) Mosaic plot of immune cell clusters, each of which is composed of Fn-positive and Fn-negative groups. Red indicates significant decrease in abundance, whereas blue indicates significant increase in abundance (|Pearson residual| > 2 for p-value < 0.05, |Pearson residual| > 4 for p-value < 0.0001, one-sided chi-square test). (d) Bulk RNA sequencing data were collected from TCGA-COAD cohort and their Fn-infection was determined by detection of Fn sequence reads. (e) Progression-free survival curves by Kaplan-Meier estimation using tumors located in the right colon of the TCGA-COAD patients for different Fn-infection status.

We investigated the impact of Fn infection on immune cell composition. Our analysis revealed significant changes in the composition of intratumor immune cells between Fn-positive and Fn-negative groups. The majority of immune cell types showed a marked decrease in abundance (*r* < −4) in the Fn-positive group (Figure 1(c), Supplementary Table S2c). Conversely, immune cell types involved in immunoglobulin (Ig)-mediated immunity, such as naive B cells, germinal center (GC) B cells, and IgG plasma cells, showed a significant increase in abundance (*r* > 4) in the Fn-positive group. These findings suggest that Fn infection may lead to substantial shifts in the intratumor immune microenvironment in CRC.

Previous research has linked Fn infection with poorer prognosis in CRC, especially in tumors located in the right colon.^5,9^ To verify the prognostic impact of Fn, we analyzed bulk RNA sequencing data from right-sided CRC tumors in The Cancer Genome Atlas Colon Adenocarcinoma (TCGA-COAD). We determined Fn status, identifying 116 Fn-positive and 63 Fn-negative patients (Figure 1(d)). To minimize bias, we excluded microsatellite instability-high (MSI-H) samples, leaving 75 Fn-positive and 46 Fn-negative patients for survival analysis (Supplementary Table S3). Consistent with prior reports, Fn-positive tumors showed worse progression-free survival, though it narrowly missed statistical significance (log-rank test, *p* = 0.052, Figure 1(e)). These findings suggest that changes in intratumor immunity may influence the prognostic effects of Fn infection in CRC.

### Development of IgA-secreting plasma cells is impeded in Fn-positive CRC

Our single-cell composition analysis revealed that IgA plasma cells occupy the highest proportion in CRC tumor samples. Furthermore, we noted a significant increase in naive B and GC B cells in Fn-positive CRC samples compared to those without Fn infection (Figure 1(c)). These B cells are typically found in the Peyer’s patches of the gut-associated lymphoid tissue (GALT), where they may be activated to differentiate into IgA plasma cells.^44^ IgA plasma cells are crucial for mucosal immunity against gut commensal bacteria, producing sIgA that binds polyreactively with the gut microbiota.^45^ This binding helps prevent pathogen colonization and invasion and facilitates bacterial neutralization. Given the importance of IgA plasma cells in gut mucosal immunity, our investigation focused on these cell types. B cells within the GALT differentiate into IgA plasma cells through interactions with other immune cells, including T cells and macrophages.^46,47^

**Figure 2.**
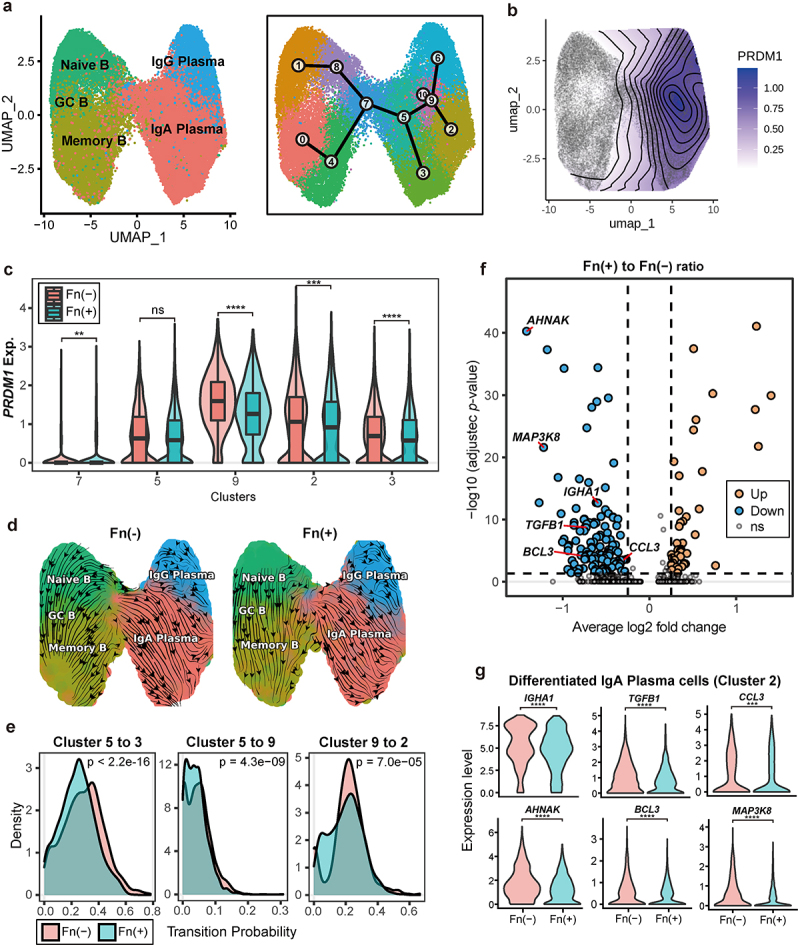
Impairment of IgA plasma cell development by Fn infection in CRC.

We employed dimension reduction techniques to reveal distinct separations of plasma cells from B cells (Figure 2(a) left). We constructed trajectories from naive B cells to IgA plasma cells, capturing transcriptional changes during differentiation (Figure 2(a) right). Expression levels of *PRDM1* (encoding Blimp-1), a well-known marker of plasma cell maturation,^48,49^ confirmed the identity of cell subsets at different stages of differentiation (Figure 2(b)). Notably, *PRDM1* expression was significantly lower in Fn-positive CRC compared to Fn-negative CRC across all differentiation stages (Wilcoxon rank sum test, *p* < 0.001; Figure 2(c)), suggesting that Fn infection hinders IgA plasma cell development. To validate the observed retardation, we conducted RNA velocity analysis, which revealed a reduced likelihood of progression along the IgA plasma cell differentiation pathway in Fn-positive CRC (Kolmogorov-Smirnov test, *p* < 0.001; Figure 2(d–e)).

(a) UMAP plot of subsets of B cells and plasma cells labeled based on cell types annotated (left), and 11 clusters obtained using the Louvain algorithm (right). Trajectory illustrating differentiation from B cells to plasma cells based on clusters. (b) Contour plot visualizing *PRDM1* expression on UMAP. (c) Boxplot comparing *PRDM1* expression in IgA plasma cells, grouped by clusters and according to the direction of differentiation, between Fn groups. The significance of differences in expression levels was estimated using a two-sided Wilcoxon rank sum test. (d) RNA velocity of B cells and plasma cells in Fn-negative (left) and Fn-positive (right) tumors. (e) Density plot of the transition probability during differentiation along the trajectory within IgA plasma cells. The statistical significance of the probability distribution was measured using the Kolmogorov-Smirnov test. (f) Volcano plot depicting the gene expression levels of differentiated IgA plasma cells (cluster 2) in Fn-negative and Fn-positive tumors. (g) Violin plots depicting differences in expression levels of genes of interest. Significance was tested using a two-sided Wilcoxon rank sum test. *P*-values were adjusted using the Family-wise Type 1 Error Rate (FWER). ns, *P* ≥ 0.05, *P < 0.05, **P < 0.01, ***P < 0.001, ****P < 0.0001.

To determine whether the observed impairment in IgA plasma cell differentiation is influenced by tumor anatomical location, we stratified the discovery cohort into left- and right-sided tumors and reevaluated *PRDM1* expression and RNA velocity dynamics. In both left- and right-sided tumors, *PRDM1* expression was consistently lower in Fn-positive samples compared to Fn-negative samples (Supplementary Figure S2a–b). Similarly, the transition probability along the IgA plasma cell differentiation trajectory was significantly reduced in the Fn-positive group regardless of tumor location (Supplementary Figure S2c–d). These findings indicate that the Fn-associated impairment of IgA plasma cell development is not restricted to a particular tumor site, but rather occurs consistently across different anatomical locations in the colon.

To investigate the functional impact of Fn infection on differentiated IgA plasma cells (cluster 2), we identified differentially expressed genes (DEGs) between Fn-positive and Fn-negative groups (Figure 2(f–g)). We confirmed the hampered development of IgA plasma cells by observing reduced *IGHA1* expression in Fn-positive group. Notably, *TGFB1* expression in IgA plasma cells also decreased in Fn-positive group. Previous studies have shown that TGF-β signaling induces differentiation from B cells to IgA plasma cells and promotes IgA secretion.^50,51^ *AHNAK* and *BCL3* were identified as regulators that enhance TGF-β signaling through interaction with the Smad-dependent canonical TGF-β signaling pathway.^52,53^ *MAP3K8* has been demonstrated to be another positive regulator of TGF-β signaling pathway.^54^ TGF-β, an immunosuppressive cytokine, has been shown to recruit TAMs and promote their immunosuppressive phenotypes, inducing M2 polarization.^55,56^ Additionally, *CCL3*, which encodes a chemokine involved in the recruitment and polarization of M2 macrophages,^57,58^ showed reduced expression in IgA plasma cells of Fn-positive group. These results collectively suggest that Fn infection reduces TGF-β production by IgA plasma cells, impeding the recruitment of M2 macrophages necessary for IgA plasma cell maturation.

### IgA induction through macrophage interaction is disrupted in Fn-positive CRC

**Figure 3.**
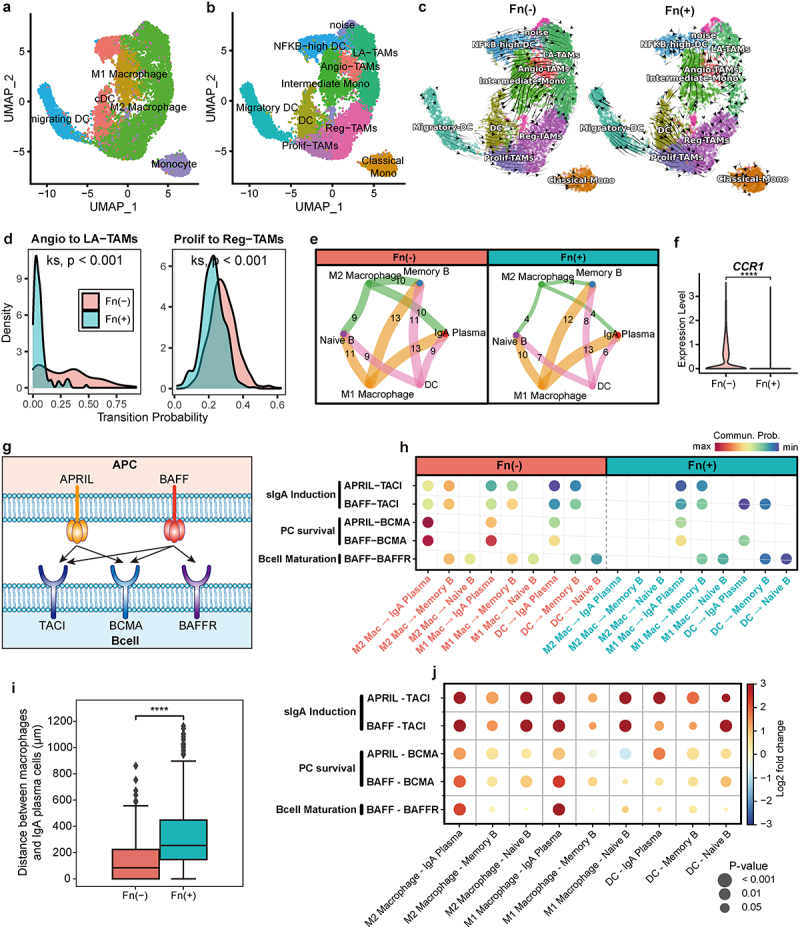
Disruption of cell-cell interactions for IgA induction in Fn-positive CRC.

Since the DEG analysis suggested a reduced capability of IgA plasma cells to recruit macrophages in Fn-positive CRC, we next examined whether Fn infection also alters macrophage functions related to their interaction with IgA plasma cells. To further specify the myeloid cell subsets, we sub-clustered populations including monocytes, macrophages, and dendritic cells (Figure 3(a)). Notably, we identified subclusters of TAMs,^59^ such as lipid-associated TAMs (LA-TAMs) and regulatory TAMs (Reg-TAMs), which are enriched in antigen processing and presentation pathways and canonical immunosuppressive M2-like pathways (Figure 3(b)). Observing cell transition patterns through RNA velocity, we noted a polarization toward these TAM clusters from surrounding cell clusters in Fn-negative tumors (Figure 3(c) left). However, in Fn-positive tumors, we observed a reduced probability of cell transitions, indicating a reduced M2 polarization of macrophages (Figure 3(c) right, 3(d)).

(a) UMAP plot of myeloid cell subsets labeled based on cell types. (b) UMAP plot of clusters of myeloid cells annotated to subtypes of Tumor-Associated Macrophages (TAMs). (c) RNA velocity of myeloid cells in Fn-negative (left) and Fn-positive (right) tumors. (d) Density plot showing the probability of transition toward cell subtypes involved in antigen processing and presentation (LA-TAMs, Reg-TAMs), from adjacent cell clusters (Angio-TAMs, Prolif-TAMs). The statistical significance of the probability distribution was determined using the Kolmogorov-Smirnov test. (e) Circle plot displaying the number of ligand-receptor interactions detected between each cell type. (f) Violin plots depicting differences in expression levels of *CCR1* in M2 macrophage. (g) Schematic illustrating ligand-receptor interactions involved in IgA plasma cell maturation. (h) Bubble plot representing cell-cell interactions (CCI) between myeloid cells, B cells, and IgA plasma cells. The color of the bubbles indicates the communication probability, with only significant interactions (permutation test, *P* < 0.001) being displayed. The known functions of each ligand-receptor pair are listed on the left side. (i) Boxplot comparing the distances between M2 macrophages and IgA plasma cells measured based on spatial transcriptome data. The statistical significance was determined using t-test. (j) Bubble plot representing CCI between myeloid cells, B cells, and IgA plasma cells based on spatial transcriptome data. The color of the bubbles represents the log_2_ fold change of the mean CCI scores estimated for Fn-negative over Fn-positive tumors. Positive values indicate higher scores in Fn-negative tumors. The size of the bubbles represents the statistical significance determined using a t-test. ****P < 0.0001.

Next, we investigated whether cell-cell interactions between B cells and myeloid cells are altered by Fn infection. Among myeloid cell subsets, M2 macrophages showed most noticeable reduction in interactions with B cell subsets, including IgA plasma cells, in Fn-positive tumors (Figure 3(e)). DEG analysis of M2 macrophages revealed the reduced expression of *CCR1*, which encodes a receptor for the CCL3 chemokine involved in the recruitment of M2 macrophages^57,58^ in the Fn-positive tumors (Figure 3(f)). Previously, we observed reduced expression of *CCL3* in IgA plasma cells of Fn-positive group (Figure 2(g)). Therefore, the reduced expression of *CCL3* in IgA plasma cells and that of *CCR1* in M2 macrophages aligns with the reduced interactions between these cell types.

Subsequently, we evaluated changes in ligand-receptor interactions between APRIL and BAFF, ligand molecules expressed on myeloid cells, and TACI, BCMA, and BAFFR, receptors expressed on B cells (Figure 3(g)). The BAFF-BAFFR interaction mediates B cell maturation.^60^ BCMA activation by either APRIL or BAFF is essential for plasma cell survival,^61^ while TACI, which interacts with either APRIL or BAFF, is critical for sIgA induction.^29^ We found that the interaction between the B-cell receptor TACI and ligands from antigen-presenting cells (APRIL, BAFF) was either absent or inhibited in the Fn-positive group (Figure 3(h)). Similarly, interactions involving BCMA, crucial for plasma cell survival, and BAFFR, involved in the maturation of B cells other than plasma cells, were also inhibited.^62,63^

To validate whether the disruption of cell-cell communications between M2 macrophages and IgA plasma cells indeed occurs in the spatial context of tumor tissue, we analyzed 10x Visium spatial transcriptomic data for CRC tumors available from public sources (Supplementary Figure S3–4). Through deconvolution analysis using the scRNA-seq data from our study, we assigned cell types to each spot of the array. We observed that the distance between IgA plasma cell spots and M2 macrophage spots was greater in an Fn-positive sample compared to an Fn-negative sample, supporting the impairment in recruiting M2 macrophages to IgA plasma cells (Figure 3(i)). Furthermore, when examining the ligand-receptor interactions measured based on their spatial coordinates, we found a significant decrease in these interactions in Fn-positive tumors compared to Fn-negative tumors (Figure 3(j)), which is consistent with the results based solely on single-cell expression information. These results collectively suggest that Fn infection disrupts cell-cell communications between IgA plasma cells and M2 macrophages, which is required for IgA induction.

### A gene network for IgA maturation is dysregulated in Fn-positive CRC

**Figure 4.**
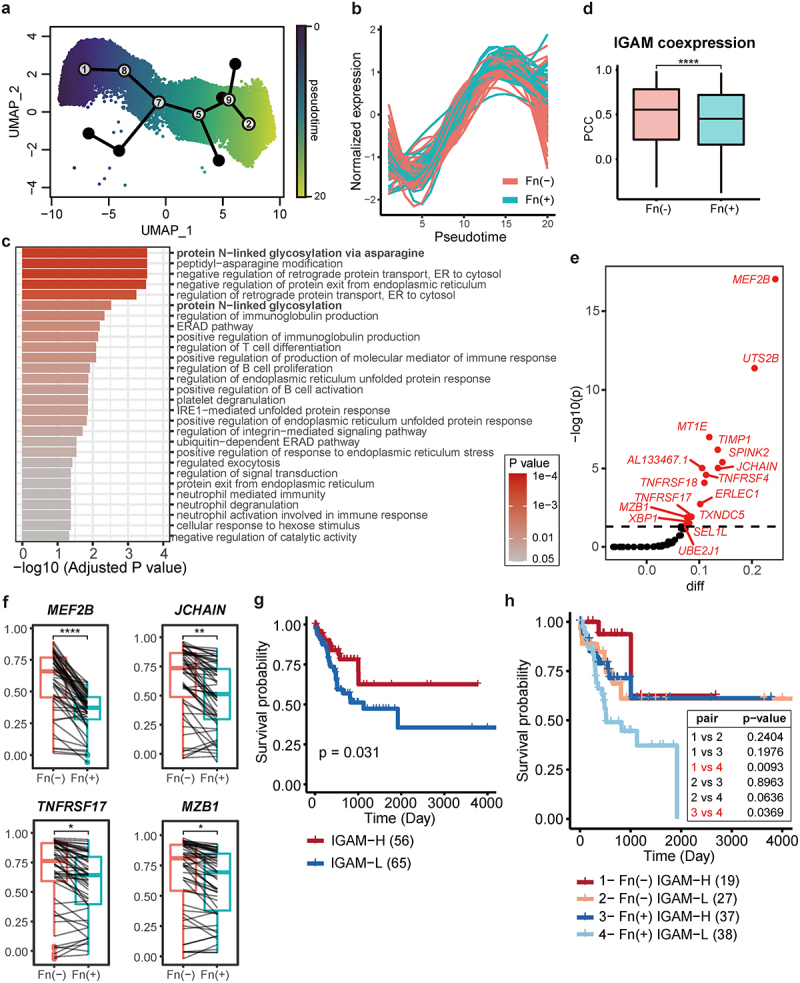
Dysregulation of a gene network for IgA maturation by Fn infection.

Disrupted IgA induction signaling due to Fn infection may lead to dysregulation of downstream molecular programs essential for secretory IgA (sIgA) production. Using single-cell transcriptome data, we analyzed the developmental process from naïve B cells to mature IgA-secreting plasma cells, focusing on cells from clusters 1, 8, 7, 5, 9, and 2 (Figure 2(a) right) for pseudotime analysis (Figure 4(a)). Then, we performed differential expression analysis along the pseudotime between Fn-positive and Fn-negative groups. We clustered genes displaying similar differential expression patterns between two Fn infection status. Among major clusters of differentially expressed genes, we chose a cluster of 55 genes including *PRDM1*, a marker for maturation of IgA plasma cell, which we termed the IgA maturation (IGAM) module (Figure 4(b), Supplementary Table S4), for the downstream analysis.

(a) UMAP visualization depicting the developmental trajectory from naive B cells to mature IgA plasma cells (identified as cluster 1, 8, 7, 5, 9, and 2). Pseudotime progression is indicated by a color gradient from dark purple (early) to bright yellow (late). (b) Graph showing changes in normalized expression over pseudotime for a cluster of 55 genes that exhibit differential expression patterns along the trajectory and between Fn infection statuses. (c) Bar graph showing significantly enriched GO biological process terms (*P* < 0.05 by Fisher’s exact test) for a cluster of 55 genes. (d) Boxplot comparing the Pearson correlation coefficient (PCC) of expressions within the IGAM gene set across Fn groups. Statistical significance was assessed using a two-sided Wilcoxon rank sum test. (e) Dot plot representing genes prioritized by a decrease in expression correlation with other network member genes upon Fn infection. Genes were sorted by the mean difference in PCC for each Fn group, adjusted using -log_10_(*p*) from a one-sided Wilcoxon rank sum test. A *p*-value cutoff of 0.05 was applied. (f) Boxplots illustrating correlation changes in significantly dysregulated genes involved in sIgA maturation and function, with statistical significance determined using a two-sided Wilcoxon rank sum test. (g) Kaplan-Meier plots estimating survival based on the IgA maturation (IGAM) gene set activity score in TCGA-COAD tumors located in the right colon. (h) Paired survival analysis conducted based on groups defined by Fn infection and IGAM gene set activity score. Statistical significance for each paired group determined using Kaplan-Meier estimation; significant pairs (*p* < 0.05) are highlighted in red. *P < 0.05, **P < 0.01, ***P < 0.001, ****P < 0.0001.

GO term enrichment analysis of the IGAM module revealed “protein N-linked glycosylation” as a critical pathway, significantly affecting sIgA-mediated mucosal immunity^64,65^ (Figure 4(c)). This glycosylation process is crucial for the interaction between IgA and the polymeric immunoglobulin receptor (plgR), which facilitates the transcytosis of dimeric IgA across mucosal epithelial cells, crucial for IgA secretion into the lumen. The glycosylated structures on IgA also directly interact with both commensal and pathogenic bacteria, suggesting that Fn infection disrupts IgA glycosylation through disturbance of the IGAM module regulation, leading to reduced IgA secretion and compromised bacterial defense in the gut lumen.

Further analysis using a network biology approach showed that coregulation within the IGAM module significantly diminished in Fn-positive groups compared to Fn-negative ones (Figure 4(d)). We prioritized genes within the module based on the decrease in expression correlation in the Fn-positive group (Figure 4(e–f), Supplementary Figure S5), revealing significant dysregulation in 16 of the 55 genes (*p* < 0.05, one-sided Wilcoxon rank sum test). These include *JCHAIN*, a protein component of sIgA responsible for the monomeric IgA joining,^66^ and *MEF2B*, a positive regulator of *JCHAIN* expression.^67^ Significant dysregulation was also observed in *TNFRSF17* (also known as *BCMA*), the receptor involved in the T cell independent IgA induction pathway, and *MZB1*, a cochaperone crucial for proper Ig heavy chain synthesis.^68^ *XBP1*, another dysregulated gene, regulates plasma cell differentiation by activating secretory machinery via unfolded protein response in endoplasmic reticulum (ER).^69–72^ Especially, *XBP1* mitigates ER stress through the upregulation of ER molecular chaperones and ER-associated protein degradation components.^73^ *ERLEC* plays a pivotal role in ER-associated protein degradation and ER homeostasis.^74^ *TXNDC5* is also a member of the protein disulfide isomerase family in ER, facilitating the cell proliferation and survival.^75^

### Stratification of Fn-positive CRC patients by IGAM activity predicts prognosis

The impact of Fn infection on clinical outcomes may vary due to differences in host genomes and intratumor immune environments. Therefore, we hypothesized that variations in the activity of the IGAM module could further stratify patients with Fn infection, potentially leading to more precise medical interventions for CRC. To explore this hypothesis, we utilized the TCGA-COAD cohort and conducted survival analysis based on IGAM activity levels, as determined by the mean expression of module genes (Methods). Our analysis revealed a significant separation between the survival curves of patients with high and low IGAM activity, irrespective of their Fn infection status. Patients with higher IGAM activity consistently showed better prognosis (Figure 4(g)). Furthermore, reduced IGAM activity was significantly associated with poorer survival in both univariate and multivariate Cox regression models, independent of Fn status, tumor stage, lymph node involvement, and microsatellite instability (Supplementary Table S5). This finding aligns with our earlier observation that the IGAM module is dysregulated in Fn-positive patients, who generally exhibit worse prognoses. Moreover, these results suggest that IGAM activity could serve as an independent prognostic biomarker in CRC.

Considering the potential association between the two prognostic biomarkers–Fn infection and IGAM activity–we developed a stratification strategy by combining these biomarkers. We divided the patients into four groups based on their Fn infection status and IGAM activity levels and compared their survival probabilities. Remarkably, we observed significantly different survival rates between two groups of Fn-positive patients differentiated by their IGAM activity levels (Figure 4(h)). Notably, Fn-positive patients with high IGAM activity exhibited survival rates comparable to those of Fn-negative patients with low IGAM activity. This suggests that enhancing IGAM activity in Fn-positive patients could potentially elevate their survival probabilities to levels observed in Fn-negative CRC patients, offering a promising avenue for tailored therapeutic strategies.

### Bacterial burden within tumor increases in Fn-positive CRC

Our single-cell analysis indicated that Fn infection was associated with diminished production of secretory IgA (sIgA), a molecule which normally binds to commensal microorganisms, preventing their adhesion and infiltration into the mucosal layer and colon tissue. Consequently, we hypothesized that the reduced production of functional sIgA in Fn-positive CRC leads to increased colonization and infiltration of gut commensals into tumor tissue, thereby inducing chronic inflammation and resulting in poor prognosis. This hypothesis aligns with a growing body of research highlighting the clinical impact of the intratumoral microbiome.^76,77^ Specifically, Fn is known to coaggregate with other bacterial species, forming biofilms that promote host invasion.^78,79^

**Figure 5.**
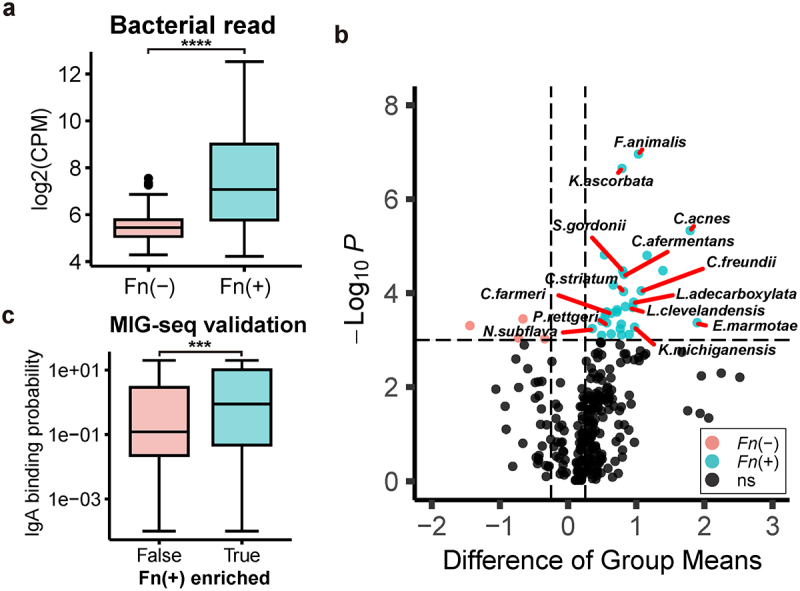
Intratumor bacteria burden increases in Fn-positive CRC.

To investigate this, we analyzed tumor bulk RNA sequencing data, using read counts aligned to human gut prokaryotic genomes as a proxy for the abundance of intratumor gut microbiota in the TCGA-COAD cohort. Our findings confirmed that intratumor bacterial abundance was significantly higher in Fn-positive compared to Fn-negative CRC cases (Figure 5(a)). Further analysis identified several bacterial species significantly increased (i.e., infiltrated) in Fn-infected tumors, including numerous pathogens or pathobionts (Figure 5(b), Supplementary Table S6).

(a) Boxplot illustrating the distribution of bulk RNA sequencing read counts aligned to human gut bacterial genomes in TCGA-COAD tumors, grouped by Fn infection status. Read counts have been normalized to the total read depth of RNA sequencing for each sample. Statistical significance was assessed using a two-sided Wilcoxon rank sum test. (b) Volcano plot depicting species enriched in each Fn infection status group. Species reported as pathogens or opportunistic pathogens are specifically labeled. Normalized read counts from each TCGA-COAD sample were sorted, and a two-sided Wilcoxon rank sum test was used to determine enrichment for each group. *P*-values were adjusted using the Family-wise Type 1 Error Rate (FWER). The plot represents species based on the difference in mean normalized read counts between Fn groups, with an adjusted *p*-value cutoff of 0.001 and a mean difference cutoff of 0.25. (c) Boxplot illustrating the distribution of IgA binding probabilities of species in TCGA-COAD tumors, grouped by the side with the higher bacterial read abundance. Statistical significance was determined using a two-sided Wilcoxon rank sum test. ***p < 0.001, ****p < 0.0001.

This increased bacterial burden within tumors may induce chronic inflammation, contributing to the poorer prognosis observed in Fn-positive CRC. A recent study assessing IgA coating levels for thousands of gut bacterial strains in healthy humans provided a metric for the IgA binding probability of each species.^80^ Our analysis found that bacterial species with increased infiltration in Fn-positive tumors also have a higher probability of binding to IgA (Figure 5(c)). These findings collectively suggest that the impairment of IgA-mediated mucosal immunity permits species typically kept in check by IgA to infiltrate the tumor, thereby exacerbating the intratumor bacterial burden in Fn-positive CRC. This mechanism likely contributes significantly to the adverse outcomes associated with Fn infection in CRC.

### Validation of Fn-associated impairment of IgA induction in an independent CRC cohort

To determine if our findings are reproducible in other CRC patients, we performed single-cell transcriptomic analysis on tumors from an independent validation cohort of 18 CRC patients (7 Fn-positives and 11 Fn-negatives) (Supplementary Table 7). This analysis identified 13 distinct immune cell types encompassing a total of 34,965 cells (Supplementary Figure S6a, Supplementary Table 8). We also examined the disruption of macrophage-mediated IgA induction by analyzing cell-cell interactions between B cells and myeloid cells (Supplementary Figure S6b). We observed notably diminished TACI-mediated interactions between B cell subsets and macrophages. Furthermore, APRIL-mediated interactions between IgA plasma cells and macrophages were reduced. These findings indicate a disruption of the interaction with macrophages necessary for IgA induction in Fn-positive tumors.

To investigate the development of IgA plasma cells further, we processed plasma cells and B cells separately (Supplementary Figure S6c). By assessing *PRDM1* expression, we distinguished mature IgA plasma cells (Supplementary Figure S6d). Using RNA velocity to measure cell transitions, we noted a significant delay in the transition to mature IgA plasma cells in the Fn-positive group, replicating our results from the discovery cohort (Supplementary Figure S6e-f). Lastly, we estimated the intratumor bacterial read count using bulk RNA-seq data from the tumor samples of the validation cohort. The analysis revealed a consistent increase in intratumor bacterial read count in the Fn-positive group compared to the Fn-negative group, although the sample size was too small for statistical significance (Supplementary Figure S6g).

In summary, single-cell and tumor microbiome analyses in an independent validation cohort reinforced our observations from the discovery cohort, confirming that the poor prognosis of Fn-positive CRC patients is linked to the impairment of IgA-mediated mucosal immunity.

### Validation of Fn-associated impairment of IgA induction across mouse models

**Figure 6.**
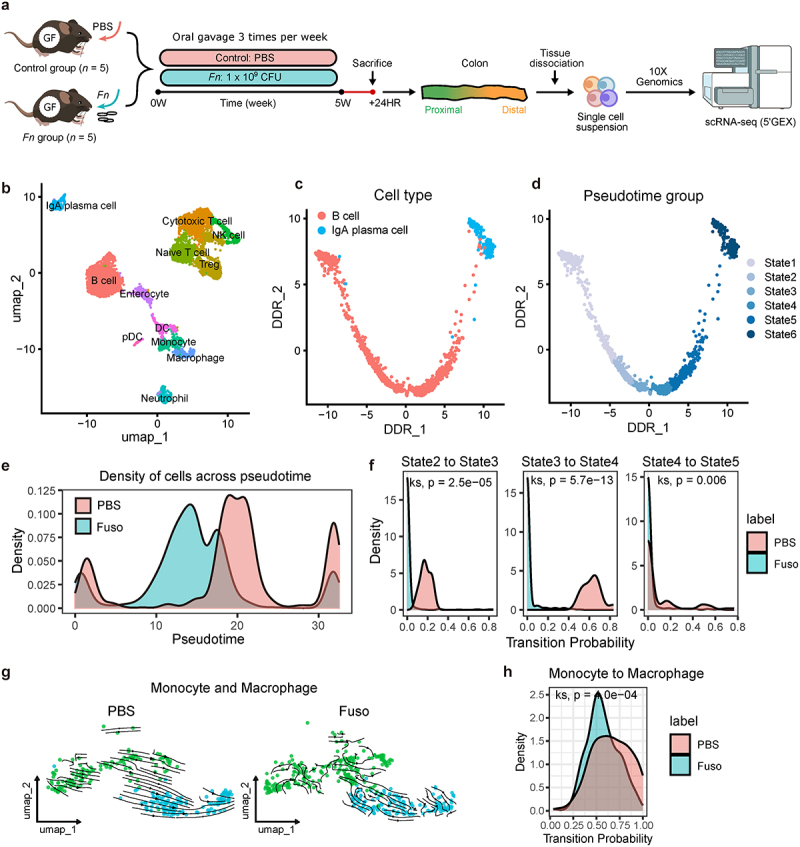
Disruption of IgA plasma cell and macrophage development in Fn-administered germ-free (GF) mice.

Although we identified an association between Fn infection and impaired IgA plasma cell development in an independent CRC cohort, causality was not established. To address this, we tested the causal role of Fn by infecting germ-free (GF) mice with Fn via oral gavage (Figure 6(a)). Experimental groups were established by administering Fn to GF mice (Fn group), while control groups received phosphate-buffered saline (PBS) (control group). Five weeks after oral gavage, the Fn group showed significantly lower normalized body weight (102.11 ± 0.89%, *n* = 5) compared to controls (111.11 ± 2.14%, *n* = 5) (Supplementary Figure S7a). Although GF mice typically exhibit an enlarged cecum due to mucus and undigested fibers,^81^ the Fn group showed reduced cecum size (Supplementary Figure S7b) and weight (Supplementary Figure S7c) compared to controls, though not statistically significant. These results indicate successful intestinal colonization by Fn.

(a) Experimental scheme: GF mice were administered phosphate-buffered saline (PBS) (*n* = 5) or *Fusobacterium nucleatum* (Fn) (*n* = 5) via oral gavage three times per week for five weeks, followed by sacrifice. Colon tissues were dissociated for single-cell RNA sequencing (scRNA-seq) analysis (b) UMAP visualization of immune cells, identifying 12 distinct immune cell types after preprocessing and batch correction. (c) Differentiation trajectories of B cells and plasma cells visualized using the DDRTree algorithm. (d) Six cell states along the pseudotime trajectory of B cell-to-IgA plasma cell differentiation. (e) Cell density distribution along pseudotime for IgA plasma cell differentiation in Fn (Fuso) and control (PBS) groups. (f) Density plot of transition probabilities between cell states along the IgA plasma cell trajectory (Kolmogorov-Smirnov test). (g) RNA velocity analysis of monocytes and macrophages from colon tissues in control (PBS) and Fn (Fuso) group. (h) Density plot of transition probability from monocytes to macrophages (Kolmogorov-Smirnov test).

scRNA-seq analysis generated single-cell transcriptome profiles for 7,470 mouse immune cells (Figure 6(b)). To examine IgA plasma cell differentiation, we constructed a trajectory from B cells to IgA plasma cells (Figure 6(c)), grouping cells along pseudotime into distinct developmental states (Figure 6(d)). The trajectory was validated by the expression dynamics of marker genes: early-state B cells (*Bach2*, *Ebf1*, *Foxp1*, *Pax5*)^82–84^ (Supplementary Figure S7d), mature B cells (*Cd37, Ce52, Cd74*, *Cd79a*)^82^ (Supplementary Figure S7e), and IgA plasma cells (Supplementary Figure S7f). Notably, cell density distributions shifted toward earlier pseudotime in the Fn group, indicating delayed IgA plasma cell differentiation (Figure 6(e)). RNA velocity analysis showed inhibited transition from early-state B cells to mature B cells (Figure 6f) and from monocytes to macrophages, essential for IgA induction (Figure 6(g–h)) in the Fn group. These findings suggest that Fn disrupts the development of both IgA plasma cells and macrophages, ultimately impairing IgA-mediated mucosal immunity.

To test the role of Fn under a more physiologically relevant setting-one that preserves both the tumor-immune microenvironment and the normal gut microbiome – we re-analyzed a publicly available single-cell RNA-seq data set from a CRC mouse model with an intact microbiota (GEO accession GSE172334). From 36,697 tumor-infiltrating cells collected from three mice gavaged orally with Fn and three gavaged with PBS, we identified 15 immune cell types (Supplementary Figure S8a). We then subclustered the B-lineage compartment, resolved a distinct germinal-center (GC) B-cell cluster, and reconstructed a differentiation trajectory from naïve/GC B cells to IgA plasma cells (Supplementary Figure 8b). In the Fn group, expression of key plasma-cell – differentiation genes – including *Tnfrsf13b* and *Tnfrsf13c*—was significantly reduced in B cells (Supplementary Figure S8c). When the PBS and Fn samples were visualized separately, the GC-to-IgA trajectory appeared less continuous in Fn-exposed mice (Supplementary Figure S8d). RNA-velocity analysis confirmed this impression: the transition probability from B cells to IgA plasma cells at the branch point was markedly lower in the Fn cohort (Supplementary Figure S8e–f). Thus, Fn impairs IgA-plasma-cell differentiation not only in germ-free mice but also in conventionally colonized animals.

## Discussion

Our analysis revealed significant alterations in the immune cell composition within tumors due to Fn infection, including the dysregulation of pathways essential for generating IgA-secreting plasma cells. While existing studies have shown that Fn infection inhibits T-cell-driven antitumor responses,^85^ our research highlights the crucial role of specific B-cell subsets in the poor prognosis of Fn-associated CRC. Notably, Fn infection hindered the development of IgA plasma cells in the colon tissue of germ-free mice and in the tumor tissue of a CRC mouse model with a conventional microbiota. Single-cell and spatial transcriptome data analysis showed disrupted ligand-receptor interactions between IgA plasma cells and M2 macrophages, resulting in impaired maturation and mucosal immunity functions of sIgA in Fn-positive CRC. Consequently, the weakened sIgA function may not properly prevent gut bacteria infiltration, leading to an elevated intratumor bacterial burden, which may adversely affect patient clinical outcomes by inducing chronic inflammation. Remarkably, we identified signature genes for IgA maturation and found that their activity can stratify Fn-positive CRC patients. Those with higher gene activity exhibit survival rates comparable to Fn-negative patients. These findings illuminate the potential for developing personalized therapies for Fn-positive CRC patients by targeting the IgA development pathway.

Patients with IgA deficiency exhibit an increased risk of several types of cancer, including gastrointestinal cancer.^86^ sIgA normally acts as the primary antigen-specific defense mechanism within the intestinal lumen, playing a crucial role in defending against enteric pathogens.^45,46^ Additionally, sIgA naturally binds to and encapsulates commensal bacteria in the gut, thereby playing an essential role in maintaining the balance between the host and its gut microbiota. Our results suggest that individuals with intact IgA function in colon tumors may have improved prognoses in Fn-positive cases. This implies that the role of sIgA in counteracting Fn infection could serve not only as a potential biomarker for early detection of colon cancer^87^ but also as a promising leverage for therapeutic strategies aimed at inhibiting Fn-induced tumor progression. These findings underscore the need for further studies to identify the specific antigens recognized by sIgA and to elucidate how sIgA neutralizes the pro-tumoral effects of Fn infection in colon cancer.

This study has some limitations. First, while we identified the causal role of Fn infection in dysfunctional sIgA production in CRC tumors, the precise molecular mechanism remains unclear. Our spatial transcriptome analysis revealed that IgA plasma cell spots were located closest to the spots containing Fn sequence reads in situ, suggesting that Fn might impair IgA plasma cells through close contact. However, further studies are needed to identify Fn-derived molecules that negatively regulate TGF-β signaling in IgA plasma cells. Second, clinical factors such as antibiotic usage and the effects of chemotherapy were not available for the TCGA-COAD cohort and therefore not included in the multivariate survival analysis. In our independent patient cohort, however, all individuals were treatment naïve and had not received antibiotics at the time of curative-intent surgery and sample collection. Additionally, the IGAM module for stratifying Fn-positive patients was not validated using prospective cohorts in this study, which should be addressed in future follow-up studies.

In summary, our findings support the notion that Fn infection hinders macrophage-mediated sIgA induction and increases tumor bacterial burden via disruption of both IgA plasma cells and macrophage. Stratifying patients based on IGAM module activity and developing strategies to restore IgA maturation hold promises for refining treatment strategies for Fn-positive CRC. This approach offers a more tailored strategy for patient care, potentially improving therapeutic outcomes.

## Resource availability

### Lead contact

Requests for further information and resources should be directed to and will be fulfilled by the lead contact, Insuk Lee (insuklee@yonsei.ac.kr).

### Materials availability

This study did not generate new unique reagents.

### Data and code availability

- Single-cell RNA sequencing data have been deposited at GEO under accession number GSE273567 and will be publicly available as of the date of publication. Currently, the data can be accessed using a secure token (szaresysfxehncj).
- The corresponding raw 16S rRNA sequencing tumor microbiome data have been deposited in the Sequence Read Archive (SRA): PRJNA1142424 and will be publicly available as of the date of publication.
- Single-cell RNA sequencing data from germ-free (GF) mice have been deposited at GEO under accession number GSE288951 and will be publicly available as of the date of publication, with temporary access available via a secure token (arefyeqwllajtgn).
- Single-cell RNA sequencing data from CRC mouse with a normal gut microbiome is publicly available at GEO under accession number GSE172334.
- This paper analyzes existing, publicly available data, accessible from Broad Genome Data Analysis Center Firehose (https://gdac.broadinstitute.org) for the TCGA cohort dataset and the DDBJ Sequence Read Archive (DRA015288) for the spatial transcriptome dataset for CRC tumors.
- Any additional information required to reanalyze the data reported in this paper is available from the lead contact upon request.

## Supplementary Material

Supplemental Material
