# Supplementary material for: Secretory IgA dysfunction underlies poor prognosis in Fusobacterium-infected colorectal cancer
Source: Gut Microbes. 2025 Jul 16;17(1):2528428. doi: 10.1080/19490976.2025.2528428 (PMC12269704; doi:10.1080/19490976.2025.2528428)
Supplement: Supplemental Material [file KGMI_A_2528428_SM0975.zip › Supplementary Figures_20250616.pdf]

# Supplementary Figures

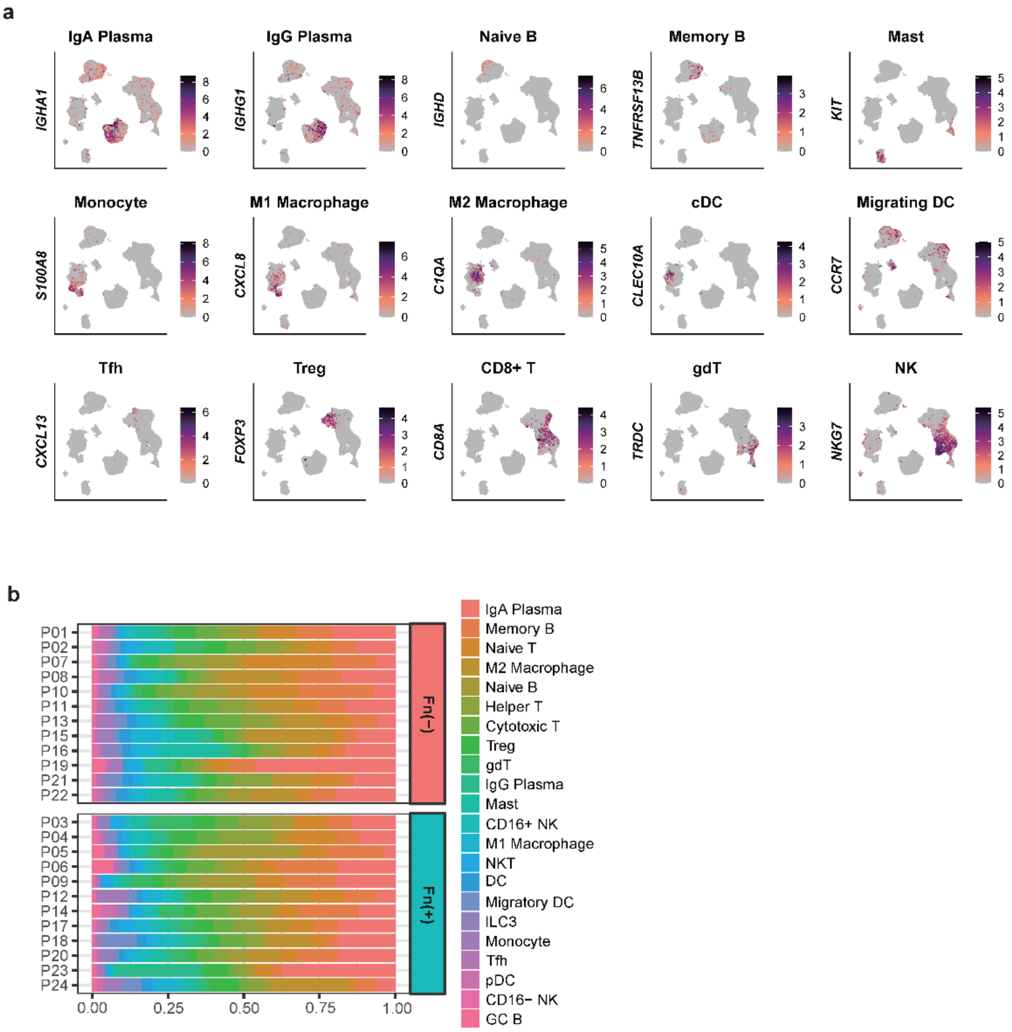


**Supplementary Figure 1. Annotation of immune cells and their composition of each tumor samples.** scRNA-seq analysis of 24 CRC tumors identified 22 immune cell types from 109,204 cells, confirming consistent cell type distribution across patients after batch correction. **a**. Cell type annotations based on the marker expression are shown in the feature maps. **b**. Composition of immune cell types for each CRC patient.

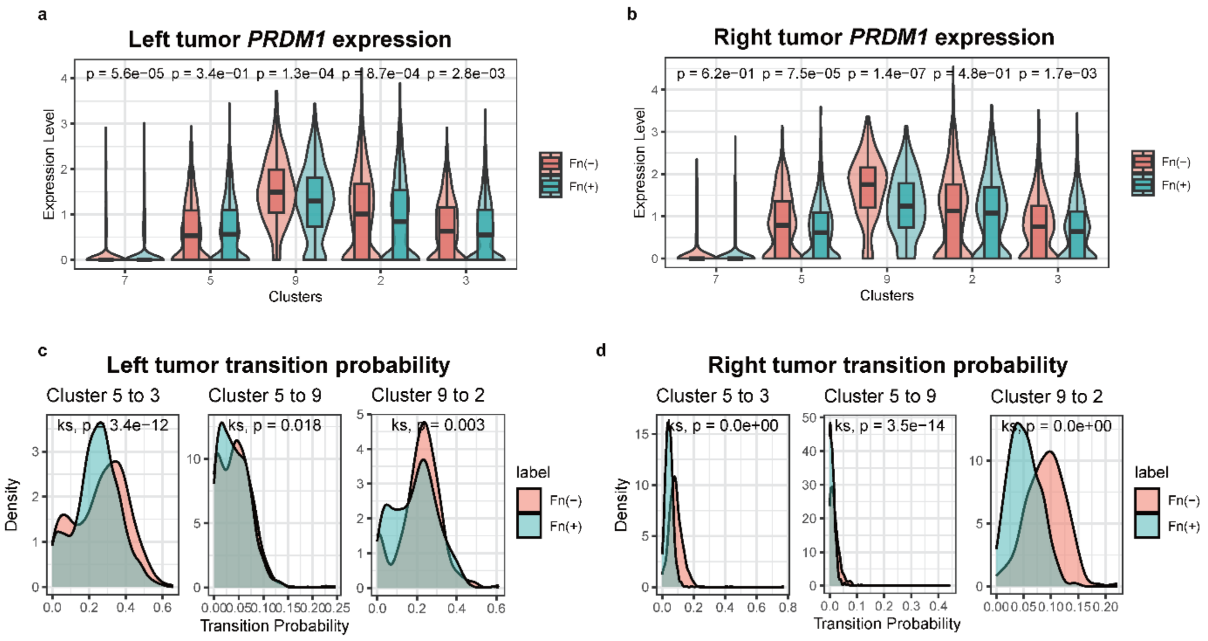


**Supplementary Figure 2. Left- and right-sided tumor analysis of IgA plasma cell differentiation.** Tumor stratification by anatomical location did not reveal appreciable differences in *PRDM1* expression or differentiation dynamics of IgA plasma cells. **a–b**. Box-and-whisker plots of *PRDM1* expression in IgA plasma cells from left-sided (a) and right-sided (b) tumors, comparing Fn-positive and Fn-negative samples across clusters ordered along the differentiation axis. *P*-values were calculated with two-sided Wilcoxon rank-sum tests. **c–d**. Density plots of transition probability along the IgA-plasma-cell differentiation trajectory for left-sided (c) and right-sided (d) tumors. Distributions were compared with the Kolmogorov–Smirnov test.

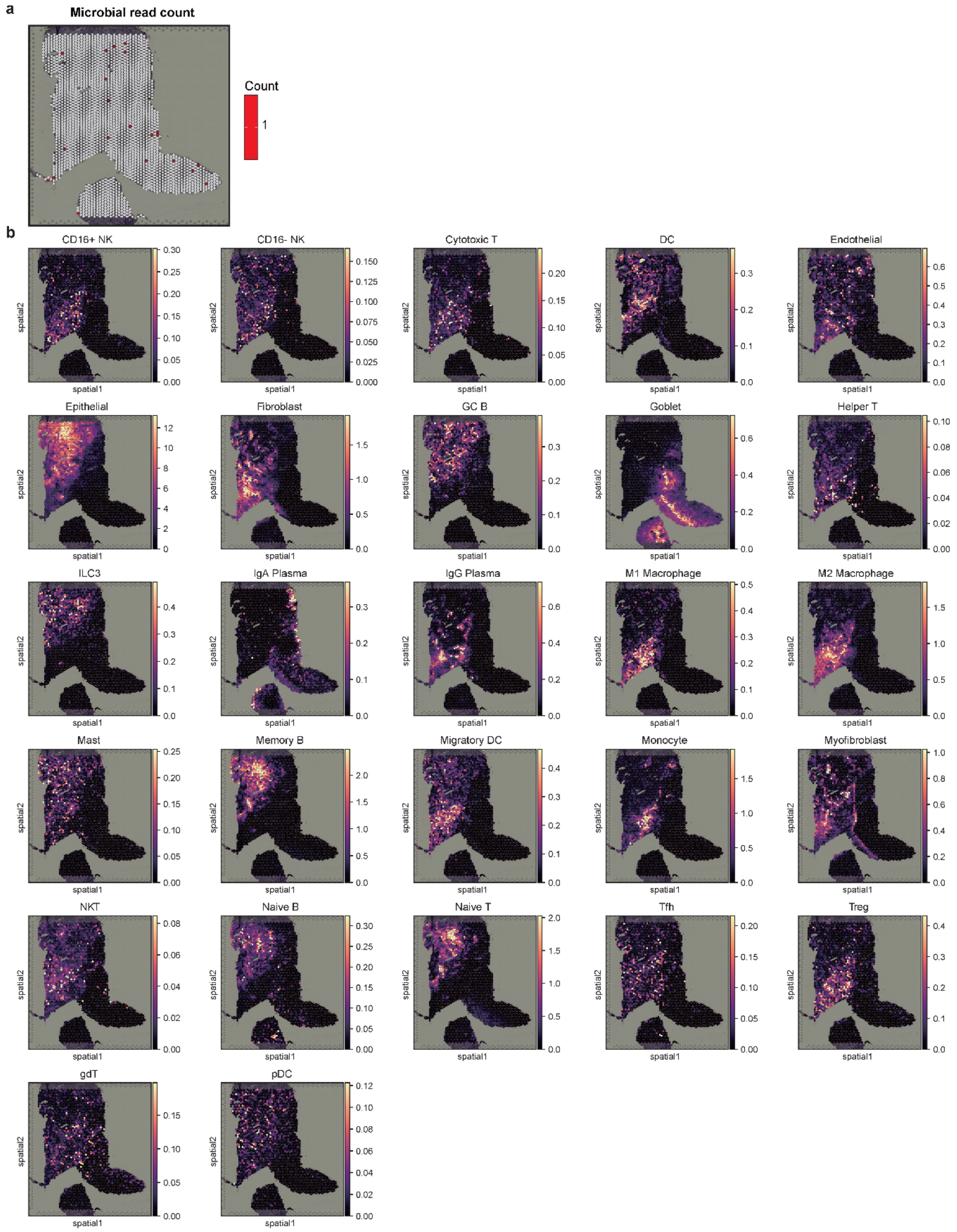


**Supplementary Figure 3. Results of the microbial read detection and cell type deconvolution of 10x Visium data from Fn-negative tumors.** Visium spatial transcriptomic data analysis validates the Fn-derived disruption of M2 macrophage–IgA plasma cell interactions within the spatial context of CRC tumors. **a.** Count of reads from the entire species in the human reference gut microbiome. **b.** Results of cell type deconvolution analysis. Each spot on the plot is colored according to the estimated abundance of different cell types.

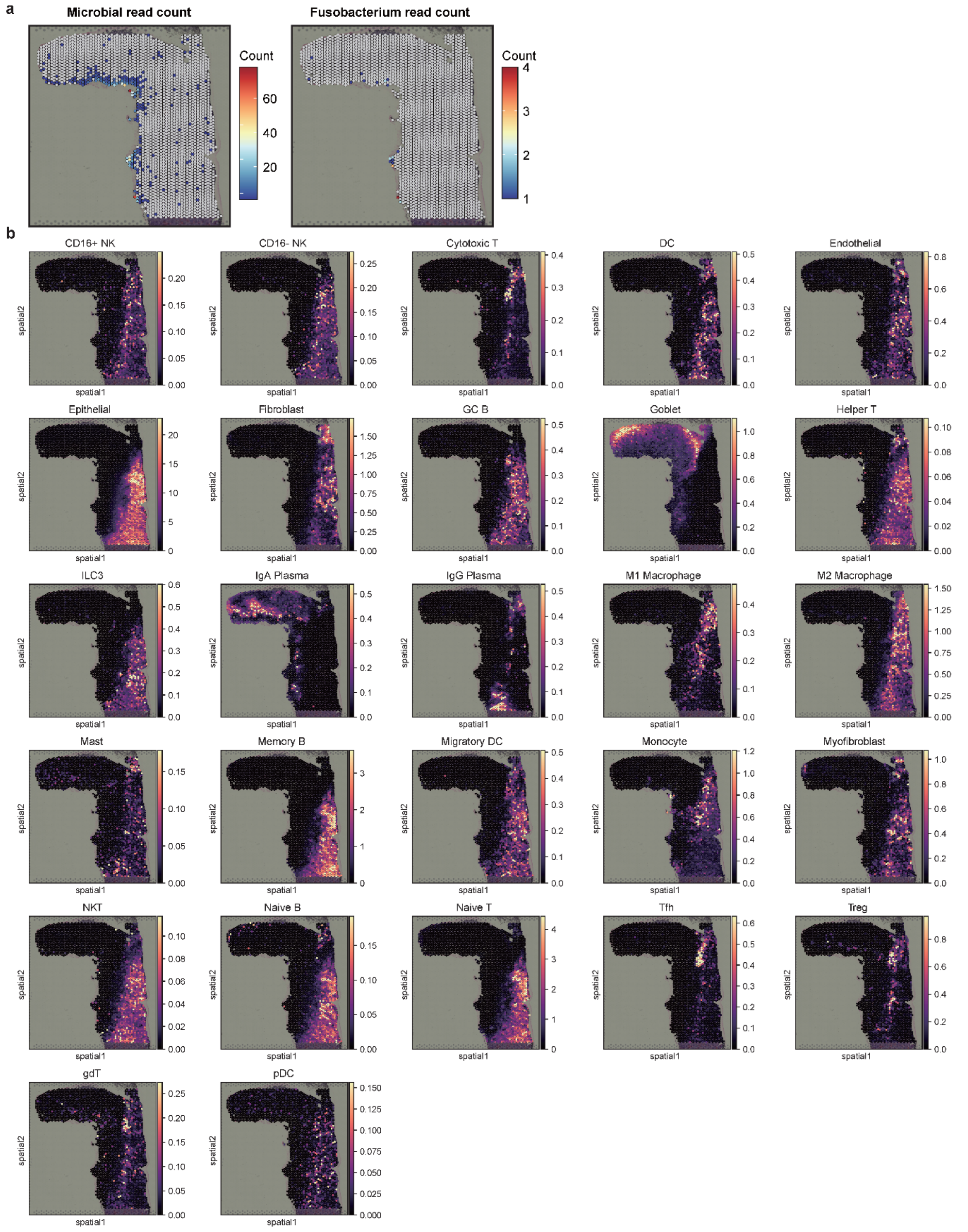


**Supplementary Figure 4. Results of the microbial read detection and cell type deconvolution of 10x Visium data from Fn-positive tumors.** Visium spatial transcriptomic data were analyzed to validate the Fn-derived disruption of M2 macrophage–IgA plasma cell interactions within the spatial context of CRC tumors. **a.** Count of reads from the entire species in the human reference gut microbiome (left) and from the Fusobacterium (right). **b.** Results of cell type deconvolution analysis. Each spot on the plot is colored according to the estimated abundance of different cell types

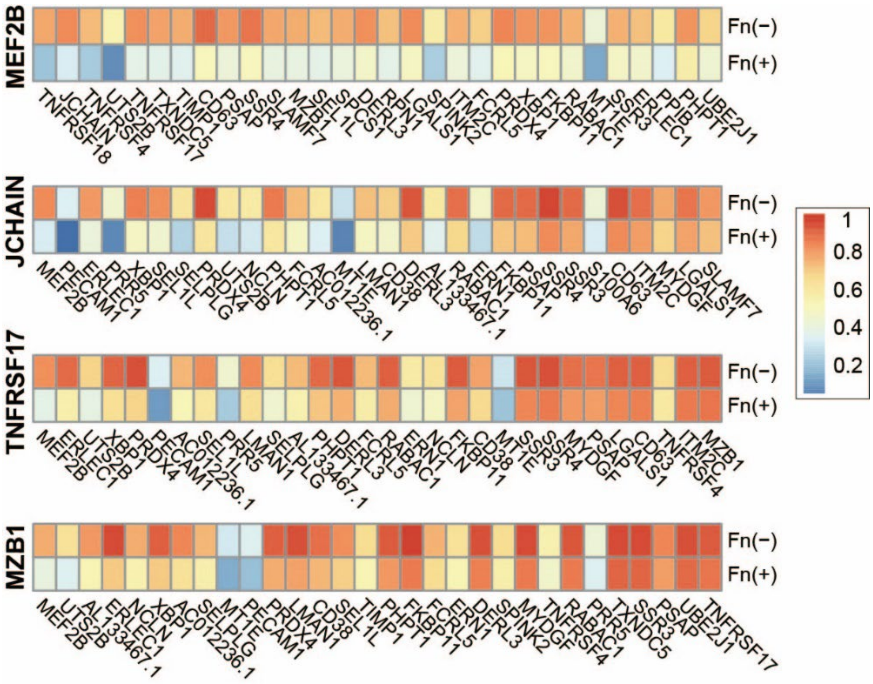


**Supplementary Figure 5. Disrupted coregulation of key IgA maturation genes in Fn-positive tumors.** Network analysis revealed a significantly reduced expression correlation among IGAM module genes in Fn-positive samples. Heatmaps display the top 30 gene-gene pairs with the largest differences in expression correlation between Fn-positive and Fn-negative groups. The correlation coefficient values are represented by color.

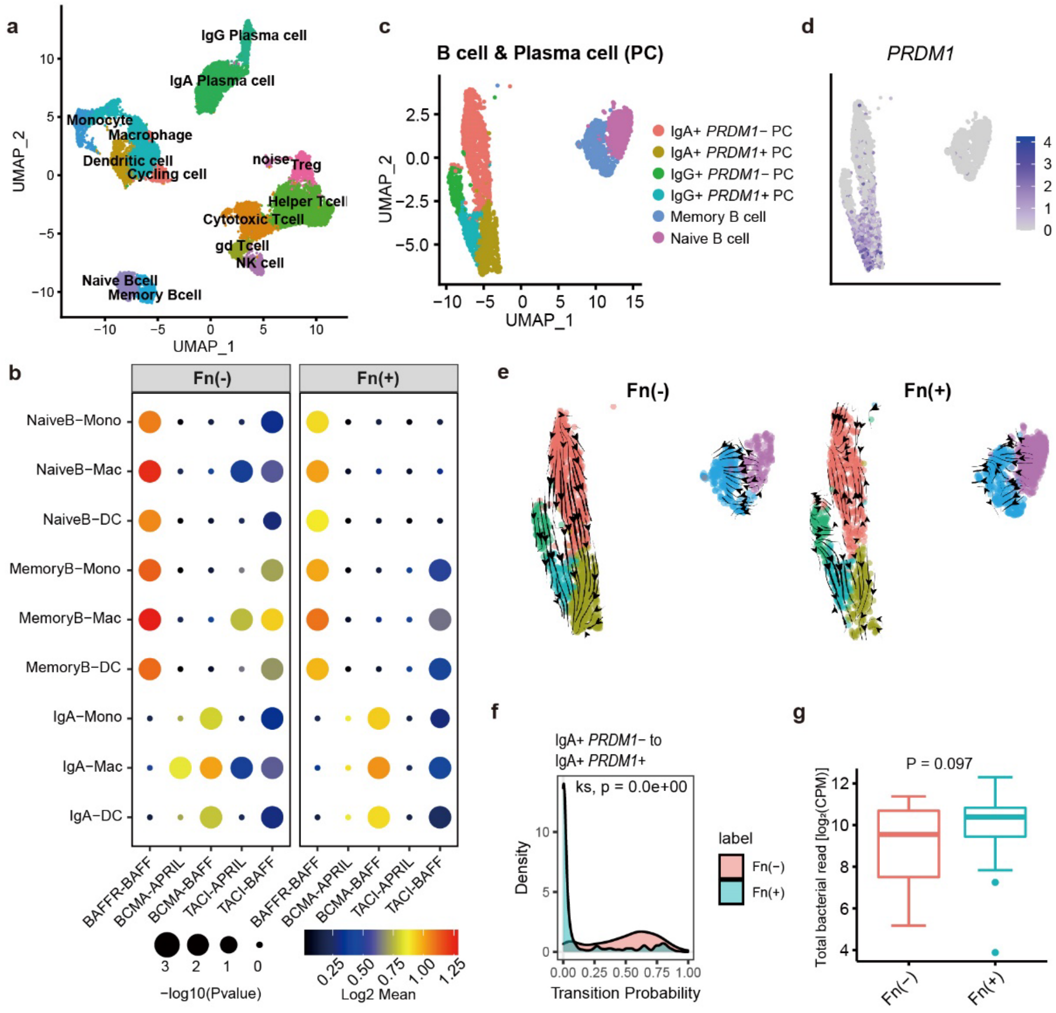


**Supplementary Figure 6. Validation cohort recapitulates reduced macrophage–B cell interactions and delayed IgA plasma cell maturation in Fn-positive tumors, confirming disrupted mucosal IgA immunity. a.** UMAP visualization of immune cells from 18 patients (11 Fn-negative and 7 Fn-positive tumors) after pre-processing and batch correction, showing a total of 13 distinct immune cell types. **b**. Bubble plot representing interactions between myeloid cells, B cells, and IgA plasma cells to examine T-cell independent IgA plasma cell maturation. The color of the bubbles indicates the total mean expression of the interacting partners. Statistical significance of each interaction was determined using permutation test. **c.** UMAP visualization of B cell and plasma cell subsets labeled based on cell types annotated at the cluster level obtained using the Louvain algorithm. **d.** Visualization of expression level of *PRDM1* expression on UMAP. **e.** RNA velocity of B cells and plasma cells illustrating both Fn-negative (left) and Fn-positive (right) tumors. **f.** Density plot depicting the transition probability during maturation of IgA plasma cells, with statistical significance of the distribution assessed using the Kolmogorov-Smirnov test. **g.** Boxplot illustrating the distribution of bacterial reads in 39 matched bulk RNA-seq sample, grouped by Fn status. Read counts have been normalized to the total read depth for each sample. Statistical significance was determined using a one-sided Wilcoxon rank sum test.

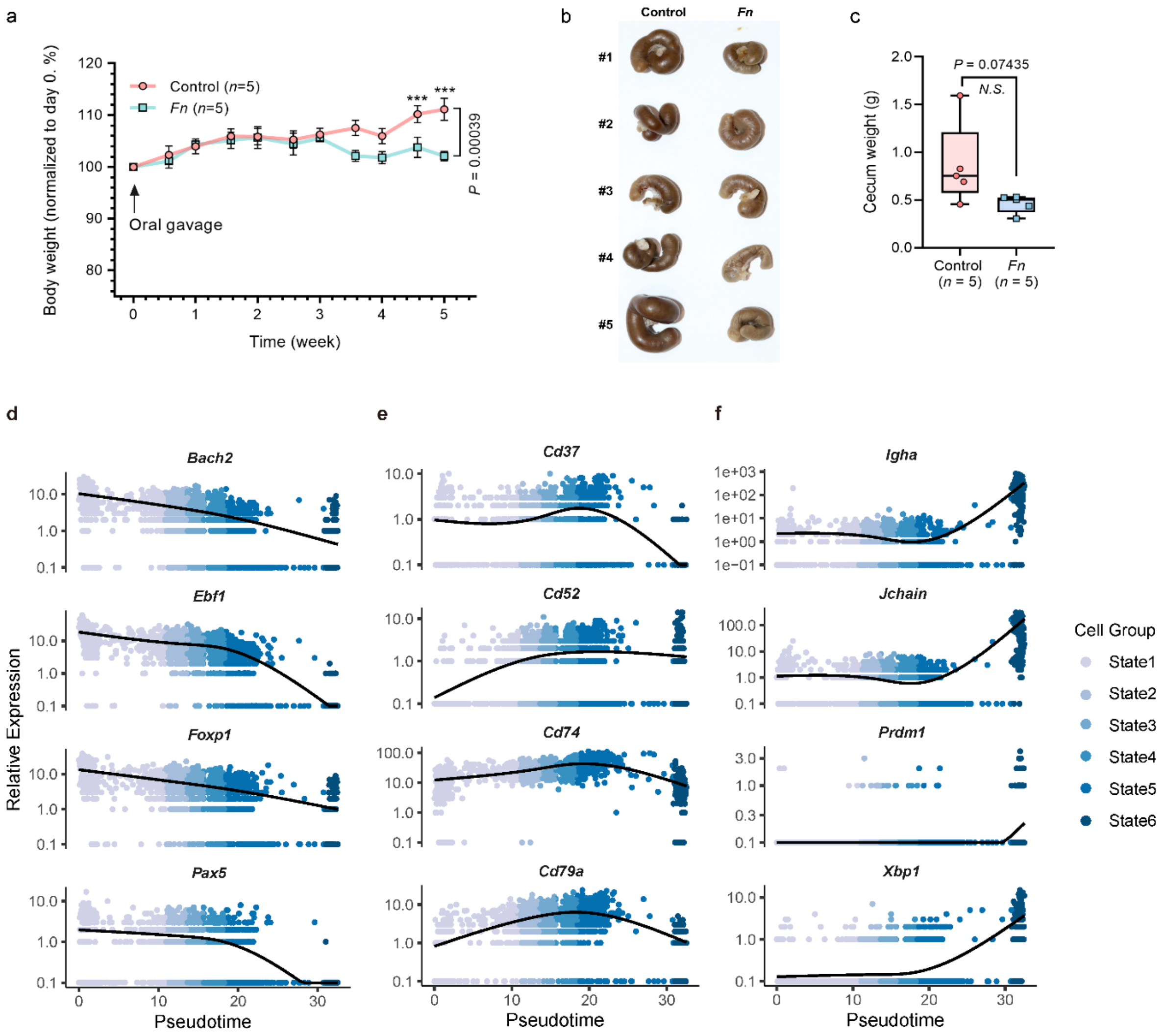


**Supplementary Figure 7. Fn-colonized germ-free (GF) mice showed reduced body weight and cecum size, supporting successful intestinal colonization and enabling assessment of Fn-driven host changes. a**. Body weight changes (percentage relative to baseline) of GF mice in control (n = 5) and Fn (n = 5) groups. Body weight was normalized to 100% on day 0. **b**. Representative images of the cecum for 5 weeks. **c**. Cecum weight (g) of GF mice in control (n = 5) and Fn (n = 5) groups. Data points are presented as the mean ± standard error (SE) for 5 mice per group. Body weight and cecum weight were analyzed using multiple Student's t-test with Bonferroni-Dunn correction (*N.S.*, not significant; *** $P < 0.001$). **d-f**. Scatter plots showing the expression of marker genes for early-state B cells (d), mature-state B cells (e), and IgA plasma cells (f), with cells ordered along pseudotime. The black solid lines represent expression patterns fitted using Generalized Additive Models (GAMs).

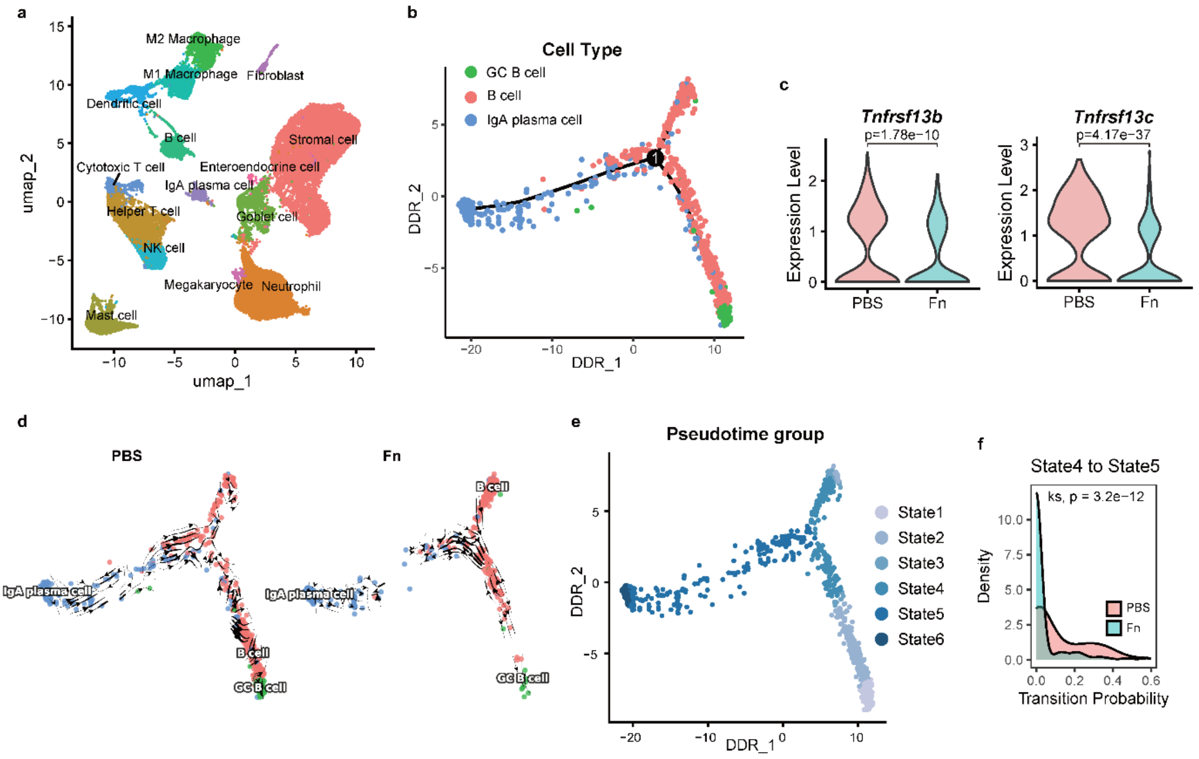


**Supplementary Figure 8. A CRC mouse model harboring a normal microbiota confirmed Fn-associated impairment in IgA plasma cell differentiation. a.** UMAP of tumor-infiltrating immune cells after pre-processing and batch correction, resolving 15 immune cell types. **b**. Differentiation trajectories of B cells and plasma cells visualized with DDRTree. **c.** Violin plots comparing the expression of genes critical for plasma cell differentiation in B cells. P-values from two-sided Wilcoxon rank sum test were adjusted for family-wise error rate. **d.** RNA velocity UMAPs of B cells and IgA plasma cells in PBS (control) and Fn (Fuso) groups. **e.** Six cell states identified along the pseudotime path from germinal-center B cells to IgA plasma cells. **f.** Density plots of transition probabilities across branching cell states; distributions were compared with the Kolmogorov-Smirnov test.
